# Proteomic analysis of the liver regulating lipid metabolism in Chaohu ducks using two-dimensional electrophoresis

**DOI:** 10.1515/biol-2022-0101

**Published:** 2022-08-17

**Authors:** Kai Ge, Zhaoyu Geng

**Affiliations:** Department of Biological and Pharmaceutical Engineering, West Anhui University, West of Yunlu Bridge, Yu’an District, Liuan, Anhui Province, 237012, China; Department of Animal Science and Technology, Anhui Agricultural University, Hefei, Anhui Province 230036, China

**Keywords:** Chaohu ducks, intramuscular fat, two-dimensional electrophoresis, proteomic analysis, lipid metabolism

## Abstract

In this study, we aimed to characterize the liver protein profile of Chaohu ducks using two-dimensional electrophoresis and proteomics. The livers were quickly collected from 120 healthy, 84-day-old Chaohu ducks. The intramuscular fat (IMF) content of the left pectoralis muscle was determined using the Soxhlet extraction method. The total protein of liver tissues from the high and low IMF groups was extracted for proteomics. Functional enrichment analysis of the differentially expressed proteins (DEPs) was conducted using gene ontology (GO) and kyoto encyclopedia of genes and genomes (KEGG). In total, 43 DEPs were identified. Functional enrichment analysis indicated that these DEPs were significantly related to four lipid metabolic processes: carboxylic acid metabolic process, ATP metabolic process, oxoacid metabolic process, and organic acid metabolic process. Three pathways correlated with lipid metabolism were identified using KEGG analysis: glycolysis/gluconeogenesis, pentose phosphate pathway, fructose, and mannose metabolism. Eight key proteins associated with lipid metabolism were identified: ALDOB, GAPDH, ENO1, RGN, TPI1, HSPA9, PRDX1, and GPX1. Protein–protein interaction analysis revealed that the glycolysis/gluconeogenesis pathway mediated the interaction relationship. Key proteins and metabolic pathways were closely related to lipid metabolism and showed a strong interaction in Chaohu ducks.

## 1 Introduction

Poultry meat is one of the most common animal food sources, accounting for approximately 30% of global meat consumption; duck products (second only to chicken products) are increasingly gaining popularity [1]. The body fat of livestock is affected by many factors, and the genetic characteristics of poultry determine the rate of fat deposition [2,3]. Therefore, selecting and breeding high-quality and lean poultry varieties is essential to reduce excessive body fat deposition [4]. Chaohu duck is a meat–egg type duck variety, originally bred around the Chaohu Lake basin in Anhui Province; it is an excellent local waterfowl variety in the nation. Chaohu duck is favored by consumers owing to its tender meat, low subcutaneous fat content, and delicious taste. However, the current feeding method for Chaohu ducks causes excessive fat deposition in their bodies owing to improved growth speed and shorter breeding cycles [5,6]. Lipid metabolism affects the growth and development of poultry and meat quality traits. Therefore, exploring the molecular mechanism of body fat deposition in Chinese native ducks and understanding how to cultivate appropriate body fat content is essential in poultry breeding.

The fat deposited in the muscles primarily affects the muscle quality, and an appropriate increase in intramuscular fat (IMF) can improve the meat quality [7] and shorten the sexual maturation time [8]. The deposition of IMF is comprehensively regulated by multiple signaling pathways [9]. The liver is the main site for the biosynthesis of sugar, fat, and protein, and these three nutrients are interconvertible through key metabolites (such as acetyl-CoA) [10]. Using two-dimensional gel electrophoresis (2-DE) proteomics, the livers from obese and lean Peking ducks were compared, and 76 proteins were found to be differentially expressed in the two types of ducks [11]. We conducted a proteomic analysis of the breast muscle of Beijing You-chicken from the embryonic stage to the early postnatal stage. There were 77 proteins in the slow type and 68 in the fast type of You-chicken [12]. Lipid production in domestic birds differs from that in mammals. Adipose tissue may play a minor role, whereas the liver may play a major role in *de novo* fatty acid synthesis. Approximately 96% of body fat is derived from liver synthesis and transport. Few in-depth studies are conducted on the correlation between the changes in IMF of Chaohu ducks and the regulatory proteins related to lipogenesis in the liver.

In this study, the liver of Chaohu ducks was selected as the study material; the proteins involved in the key regulation of fat generation, transport, and metabolism were screened through 2-DE, which provided a reference for studying the molecular mechanism of fat metabolism of Chaohu ducks and poultry and identifying candidate proteins for the genetic improvement of the quality traits of Chaohu duck meat.

## 2 Methods

### 2.1 Animals and feeding

In total, 500 healthy male ducks that were 1-day old were randomly selected from the original population of Chaohu ducks. They were bred under the same environmental conditions at Anhui Yongqiang Agricultural Science and Technology Co., Ltd (Anqing City, China). These ducks freely consumed basal pellet feed and water. At 21 days of age, 180 drakes were selected according to their body weight; they were fed for 84 days in separate duck sheds (60 ducks per shed). The indoor temperature of the duck house was 25 ± 2°C and the relative humidity was 70.5 ± 8.0%. During the entire feeding period, the basic feed of Chaohu ducks was formulated according to the nutritional standard of meat ducks of the China Agricultural Standard (Standard number: NY/T 2122-2012). All methods were carried out in accordance with the relevant guidelines and regulations and were reported in accordance with the ARRIVE guidelines (https://arriveguidelines.org) for reporting animal experiments.


**Ethical approval:** The research related to animal use has been complied with all the relevant national regulations and institutional policies for the care and use of animals. All experimental protocols were approved by the Institutional Animal Care and Use Committee of the Anhui Agricultural University, Hefei, China, under permit No. ZXD-P20140809.

### 2.2 Group design and sample collection

Chaohu ducks aged 84 days were fasted for 12 h and weighed. Based on the average body weight, 40 ducks were selected from each cage, and 120 ducks were selected for sample collection. The ducks were slaughtered using electric shock. Twenty grams of the left pectoral muscle was collected and stored at −20°C for IMF determination. Five grams of the liver was collected, stored in liquid nitrogen, and transferred to −80**°**C for protein extraction. The results of IMF content and statistical analysis of Chaohu ducks have been reported in our previous study [2]. The Chaohu ducts were divided into two groups according to the distribution rule of IMF content in breast muscle: extremely high group (CH) and extremely low group (CL). Three liver samples were collected from the CH and CL groups.

### 2.3 Extraction and concentration determination of total liver protein

Total protein was extracted from the liver samples using a mammalian proteome kit (Focus™ CAT# 786-246, G-Biosciences, Inc., USA) according to the manufacturer’s instructions. Briefly, 200 mg of the liver sample was added to 500 μL of protein solubilization buffer and homogenized for 4 min at 4°C at intervals of 10 s. The homogenate was centrifuged at 20,000×*g* for 30 min at 20°C. Subsequently, the supernatant was transferred to a clean tube and stored at −80°C until use. The total protein concentration was determined by the Bradford method (P0006C, Beyotime Biotechnology Co., Ltd, China) using an automatic enzyme label analyzer (iMark 168-1002XC, Bio-Rad, Inc., USA).

The proteins were pretreated for desalination using the acetone precipitation method before conducting 2-DE. Briefly, the protein solution was mixed with acetone at a volume ratio of 1:4 and incubated at −20°C for 12 h. Next, the mixture was centrifuged at 12,000×*g* for 10 min. After discarding the supernatant, the precipitant was air-dried at 20°C and was dissolved in a 10 mL buffer (same as the 2-DE loading buffer I) containing 4.805 g urea, 0.4 g CHAPS, 0.098 g DTT, 50 μL Bio-Lyte, 10 μL bromophenol blue, and ultrapure water. Desalted protein was used in the 2D test.

### 2.4 Separation of total protein from samples

The main reagents for 2-DE test were prepared, including immobilized pH gradient (IPG, pH 3–10, 7 cm, 163–2,000, Bio-Rad Inc., USA), hydrated loading buffer (I), tape balance buffer (I), tape balance buffer (II), 12% SDS-PAGE polyacrylamide gel, and Coomassie Bright Blue R-250 staining solution.

(1) Isoelectric focusing electrophoresis (IEF): First, the sample protein (300 μg) was added to the hydration loading buffer (I) to prepare a protein-loading liquid system with a concentration of 3 mg/mL. The protein-loading liquid was added to the focusing tray, and the IPG tape was placed. The focusing plate was placed in an IEF apparatus (Protean IEF, Bio-Rad Inc., USA), and the isoelectric focusing program was set (Table 1). After electrophoresis, the tape was removed, and a tape-balancing process was performed.
(2) SDS-PAGE electrophoresis: The 1× buffer solution was added to a vertical electrophoresis tank (Mini-PROTEAN Tetra cell, Bio-Rad Inc., USA). The balanced IPG strips were carefully transferred to the long glass plate in the interlayer to prepare the gel electrophoresis device. Electrophoresis was performed at 70 V. After the bromophenol blue indicator was completely separated from the IPG tape and concentrated in a line (approximately 20 min), the voltage was increased to 120 V, and the electrophoresis was continued for approximately 1 h.
(3) Dyeing and decolorization of gels: The SDS-PAGE gel was transferred to a decolorization tray, stained with a Coomassie Bright Blue R-250 stain solution, and decolorized using a decolorization solution.

**Table 1:** Procedure setting by IEF

Step	Voltage (V)	Gradient	Time (h/Vhr)	Function
Hydrating	50	Gradually	14	Active hydration
S1	100	Linear	0.5	Desalination
S2	250	Linear	1	Desalination
S3	500	Linear	1	Desalination
S4	1,000	Linear	1	Desalination
S5	2,000	Linear	1	Desalination
S6	3,000	Linear	1	Desalination
S7	4,000	Linear	3	Desalination
S8	4,000	Rapid	20,000	Focusing
S9	500	Rapid	Any time	Protection

### 2.5 Gel scanning and image analysis

According to the operating procedures of a calibrated optical densimeter (GS-900™, Bio-Rad Inc., USA), the gel stained with Coomassie Bright Blue was scanned, and the image information regarding three samples in the CH group and three samples in the CL group was obtained. According to the operating procedure of PDQuest 2-DE analysis software (V8.0.1) from Bio-Rad, the abundance of protein spots was analyzed on the scanning maps of each sample in the CH group and CL group, and the difference in protein spots between the two groups was screened. The analysis parameters were as follows: low quantitative value, 20; protein spot sensitivity, 15; scale size, 3; minimum peak value, 4,000; *t*-test, 95% confidence interval. The gel spots with a difference ratio of optical density (OD) ≥1.5 were identified as differentially expressed protein (DEP) spots.

### 2.6 Enzymatic hydrolysis and mass spectrometry analysis of differential protein gels

According to the image analysis data, gel points at the same position in the two groups of samples were cut and placed in a new centrifuge tube.

The gel point was rinsed thrice with ultrapure water and decolorized. Each centrifuge tube was treated with 5 μL trypsin (0.01 μg/μL) and placed on a 384-well stainless-steel plate. These samples were analyzed using mass spectrometry (MALDI-TOF-MS Analyzer 5800, AB Sciex Inc., USA). The original MS files collected by mass spectrometer were processed, retrieved, analyzed, and identified by Mascot software (V2.5.1), with reference to the NCBI-*Anas platyrhynchos* protein database (https://www.uniprot.org/).

### 2.7 Bioinformatics analysis of DEPs

The DEPs identified through mass spectrometry (*P* ≤ 0.05) were analyzed using bioinformatics, including gene ontology (GO) enrichment analysis, kyoto encyclopedia of genes and genomes (KEGG) pathway analysis, and protein–protein interaction (PPI) analysis. Based on the QuickGO database (http://www.geneontology.org) and the KEGG database (https://www.kegg.jp/), the significantly enriched GO terms and KEGG pathways were screened at *P* ≤ 0.05. A PPI pathway map was developed by mapping the DEPs and their related pathways into STRING online database (http://string.embl.de).

## 3 Results

### 3.1 Total protein concentration of the sample

**Figure 1:**
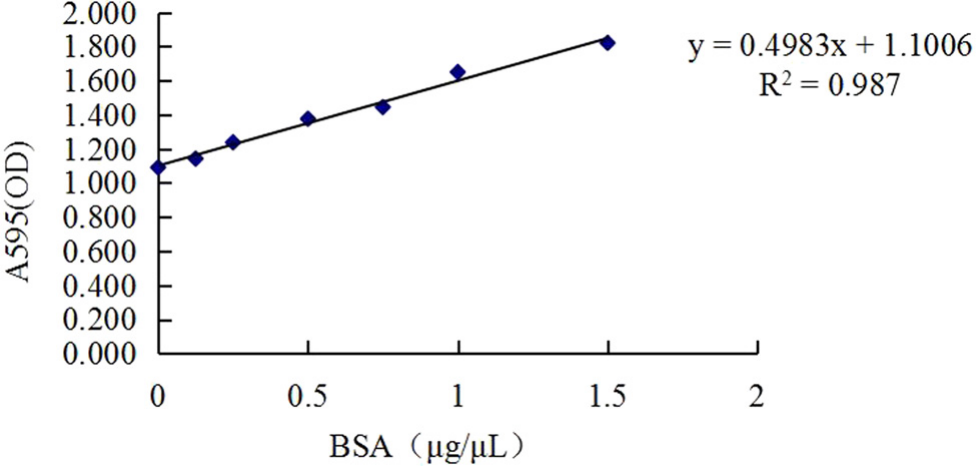
Standard Bradford curve for protein contents. Note: The *x*-axis denotes BSA, bovine serum albumin concentration (μg/μL); *y*-axis denotes OD value at A595, absorbance of the microplate reader at a wavelength of 595 nm.

According to the standard protein curve of Bradford’s method (Figure 1), protein concentrations of six samples in the CL and CH groups were calculated, which ranged from 21.64 to 27.24 μg/μL, and the total protein content of each sample was more than 6,492 μg (Table 2).

**Table 2:** Concentration of liver protein sample from Chaohu ducks

Sample	OD	Concentration (μg/μL)	Volume (μL)	Protein weight (μg)
CL-1	2.30	24.13	300	7,239
CL-2	2.25	23.15	300	6,945
CL-3	2.18	21.64	300	6,492
CH-1	2.46	27.24	300	8,172
CH-2	2.35	25.03	300	7,509
CH-3	2.29	23.99	300	7,197

### 3.2 Gel analysis of the total protein in CH and CL groups

**Figure 2:**
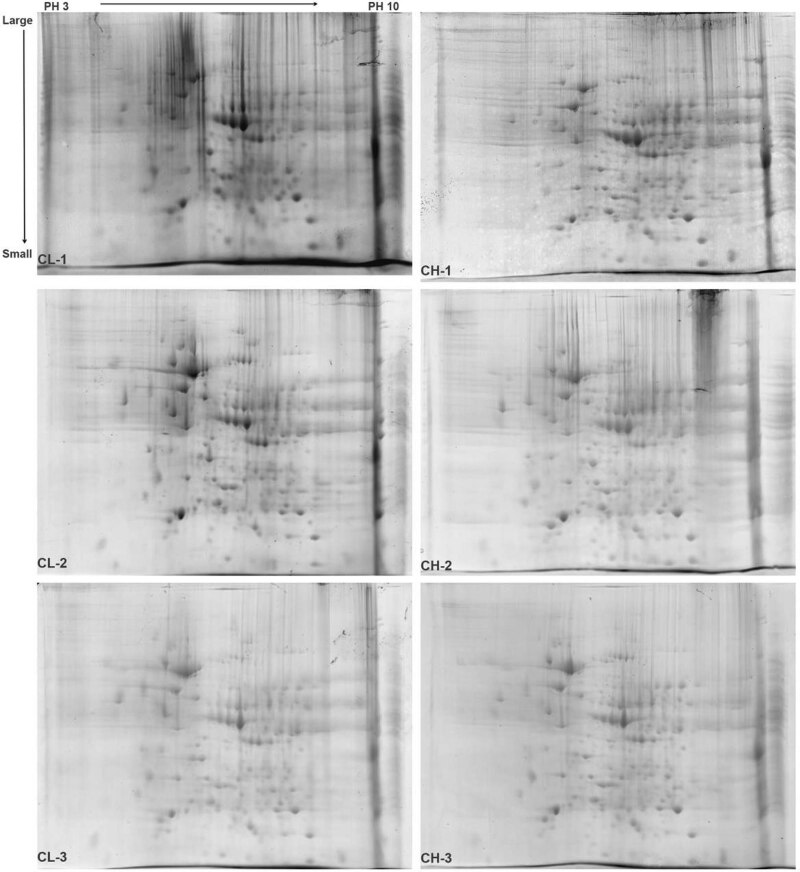
2-DE gel diagram of liver protein samples in Chaohu ducks. Note: PH 3 → 10 is the isoelectric point of the protein from the IPG strip, which gradually increases from left to right. Large → Small is the molecular weight of the protein that gradually decreases from top to bottom.

**Figure 3:**
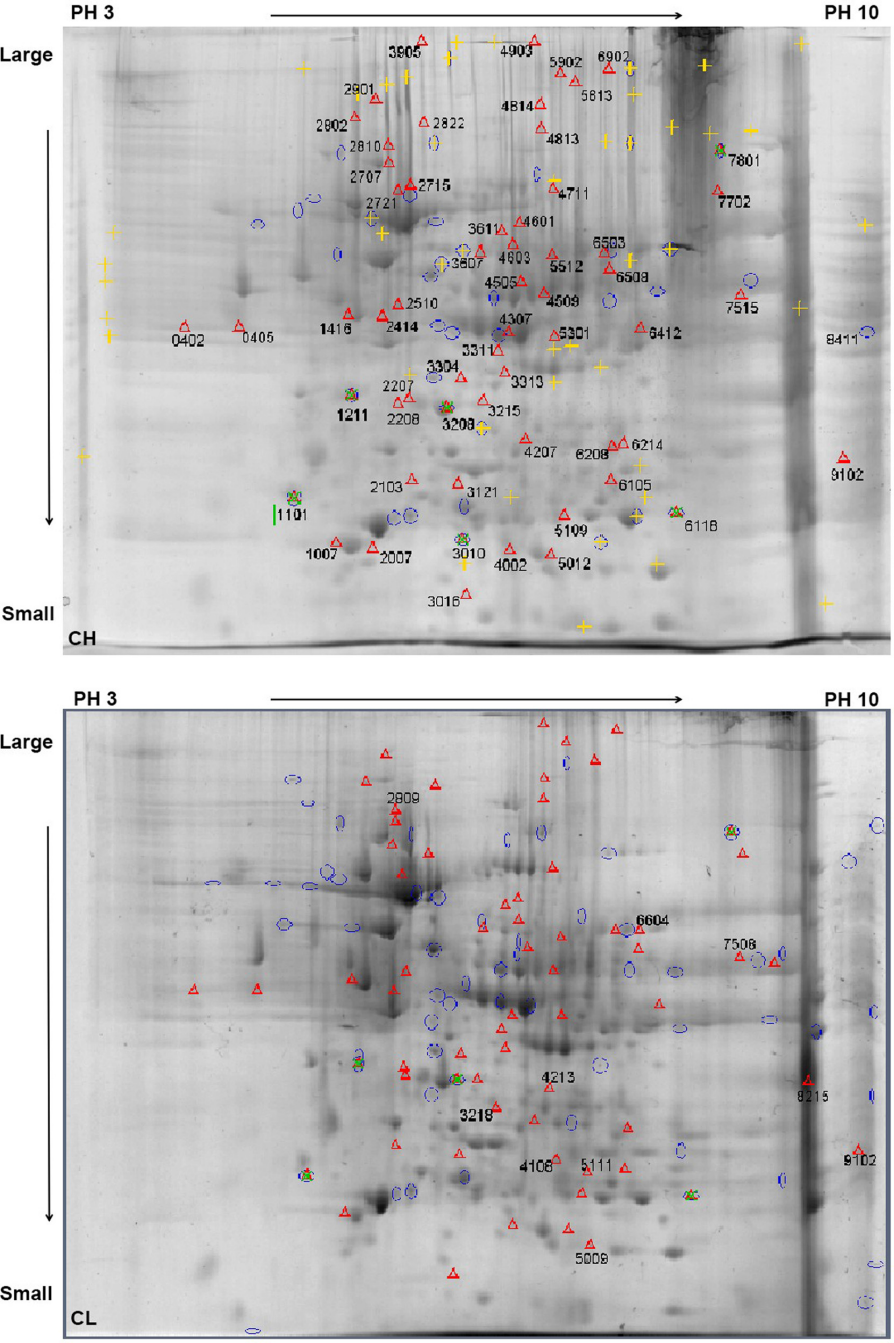
Alignment of differential protein points from Chaohu duck protein samples in 2-DE gel. Note: Red triangle mark and serial number “Δ0402” on the gel are the different protein points of the CH group compared with the CL group. pH 3 → 10 is the isoelectric point of the protein from the IPG strip, which gradually increases from left to right. Large → Small is the molecular weight of the protein gradually decreasing from top to bottom.

The total liver proteins of the CH and CL groups were separated using 2-DE, and the protein separation maps in each group showed repetitions, with clear protein spots and few background impurities (Figure 2). The PDQuest analysis software was used for image comparison and analysis. There were 68 differential protein spots in the total protein map of liver samples between the CH and CL groups. The screened differential protein spots were labeled on the gel map of the CH and CL groups (Figure 3).

### 3.3 Identification of differential protein spots using mass spectrometry

The 68 DEPs were digested with trypsin and identified using mass spectrometry. According to the NCBI-*Anas platyrhynchos* protein database, 43 DEPs, including fructose bisphosphate aldolase B (ALDOB), were screened. All the DEPs were upregulated proteins (Table A1).

### 3.4 GO enrichment analysis of DEPs

The 43 DEPs were mapped to the GO database for enrichment analysis. The significantly enriched GO functional items included 598 biological processes (1,448 in total), 77 cell components (214 in total), and 118 molecular functions (256 in total).

**Figure 4:**
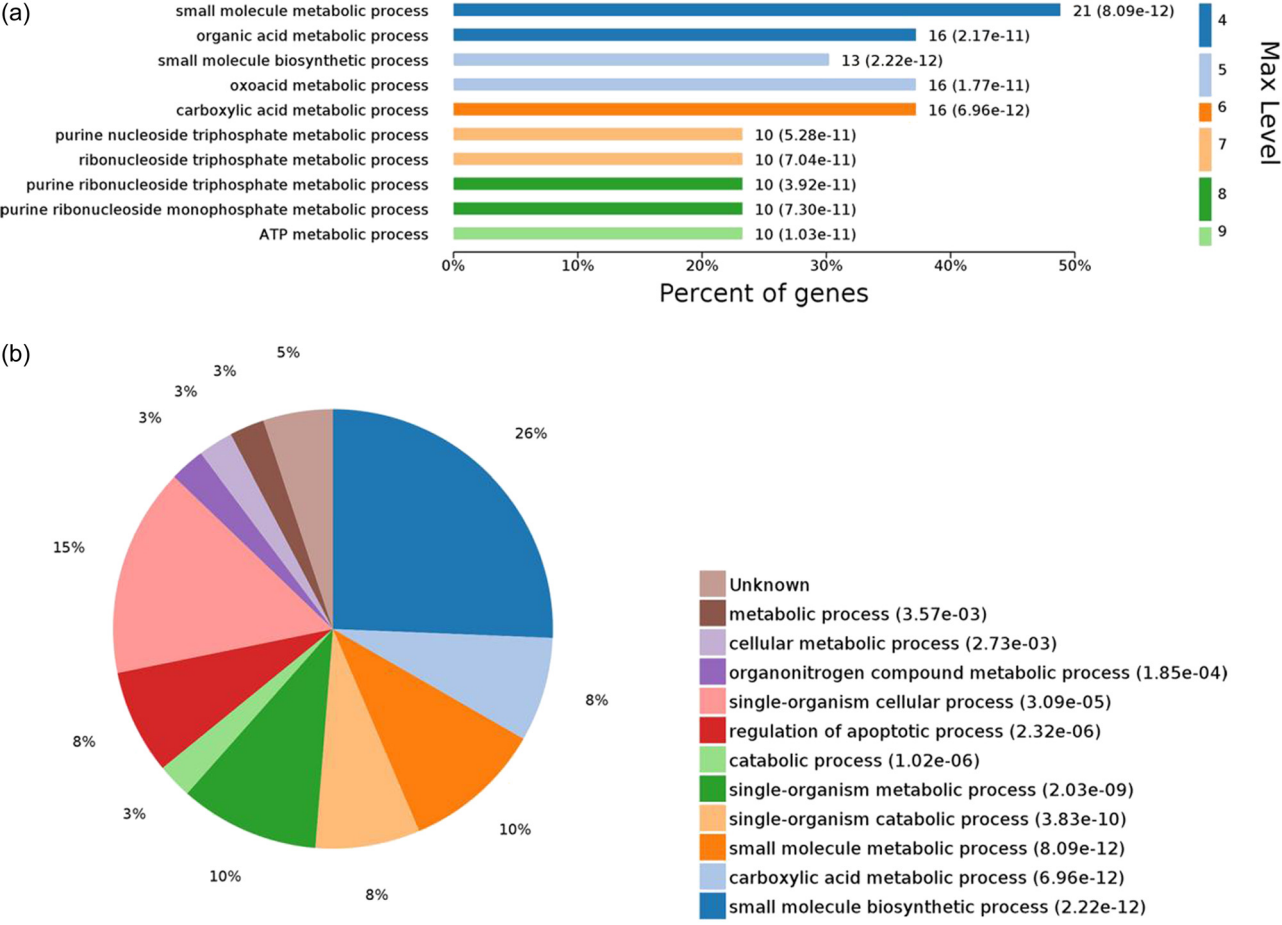
GO enriched biological process terms with statistically significant differences. Note: Map (a) shows the top ten biological processes for statistically significant differences; *y*-axis denotes the difference order at the maximum level terms; *x*-axis denotes the percentage of enriched proteins in each term. Map (b) shows the proportion distribution of all biological processes, with *P ≤* 0.05.

Considering the top ten biological processes, the small molecule biosynthetic process (*P* = 2.22 × 10^−12^) had the highest statistically significant difference. Four metabolic processes were significantly related to lipid metabolism, including carboxylic acid, ATP, oxoacid, and organic acid metabolic processes (Figure 4). Additionally, eight DEPs were found to be significantly enriched: ALDOB, triosephosphate isomerase (TPI1), alpha-enolase (ENO1), glyceraldehyde 3-phosphate dehydrogenase (GAPDH), regucalcin (RGN), peroxiredoxin-1 (PRDX1), glutathione peroxidase (GPX1), and stress-70 protein family mitochondria (HSPA9). These DEPs were simultaneously involved in multiple GO functional processes related to carbohydrate and lipid metabolism, suggesting that these proteins are closely related to fat deposition in Chaohu ducks.

### 3.5 KEGG pathway enrichment analysis of DEPs

**Figure 5:**
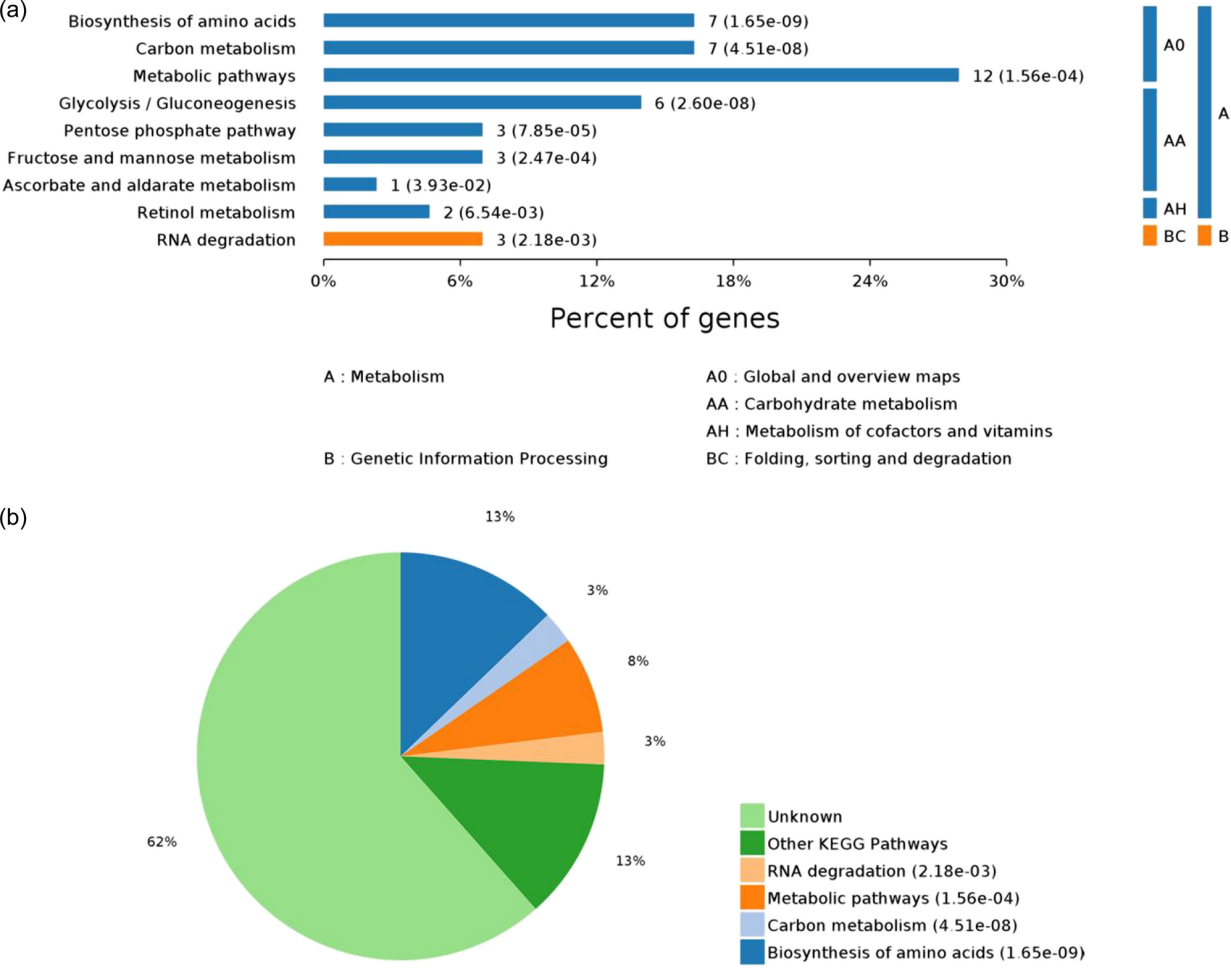
Pathways with significant differences in KEGG enrichment. Note: Map (a) shows all KEGG enriched pathways with *P* ≤ 0.05; *y*-axis denotes the pathway classification and name; *x*-axis denotes the percentage of enriched proteins in each pathway. Map (b) shows the proportion distribution of pathways with *P* ≤ 0.05.

The above-mentioned 43 DEPs were significantly enriched in nine pathways (*P*-value ≤ 0.05), including the biosynthesis of amino acids, glycolysis/gluconeogenesis, and pentose phosphate pathway. Among these, the biosynthesis of amino acids showed the highest statistically significant difference (*P* = 1.65 × 10^−09^). The following three pathways were related to lipid metabolism: glycolysis/gluconeogenesis (*P* = 2.60 × 10^−8^), pentose phosphate pathway (*P* = 7.85 × 10^−5^), and fructose and mannose metabolism (*P* = 2.47 × 10^−4^) (Figure 5). Six DEPs were significantly enriched: ALDOB, ENO1, GAPDH, TPI1, RGN, and HSPA9. In addition to lipid metabolism pathways, these DEPs were also involved in the biosynthesis of amino acids, carbon metabolism, RNA degradation, ascorbate, and aldarate metabolism. Thus, metabolic pathways of carbohydrates, lipids, and proteins are interrelated and responsible for fat deposition in the body.

### 3.6 PPI of DEPs related to lipid metabolism

Through the bioinformatic statistical analysis, three key metabolic pathways and eight key DEPs were found to play an important role in the regulation of lipid metabolism. The three key metabolic pathways included glycolysis/gluconeogenesis, pentose phosphate pathway, and fructose and mannose metabolism. The eight key proteins were ALDOB, GAPDH, ENO1, RGN, TPI1, HSPA9, PRDX1, and GPX1 (Table 3).

**Table 3:** Lipid metabolism related regulatory proteins information

Gene name	Uniprot-ID	Log_2_ fold change	Identity (%)	*E*-value	InterPro description
PRDX1	P0CB50	3.063502942	98.99	5.00 × 10^−145^	Peroxiredoxin-1
HSPA9	Q5ZM98	1.555816155	98.69	0	Stress-70 protein mitochondrial
TPI1	P00940	1.469885976	99.6	0	Triosephosphate isomerase
ENO1	P51913	1.189033824	97.24	0	Alpha-enolase
GAPDH	P00356	1.163498732	99.1	0	Glyceraldehyde-3-phosphate dehydrogenase
ALDOB	P07341	0.831877241	94.15	7.00 × 10^−117^	Fructose-bisphosphate aldolase B
GPX1	R4GH86	0.799087306	100	4.00 × 10^−114^	Glutathione peroxidase
RGN	Q9I923	0.782408565	88.76	7.00 × 10^−179^	Regucalcin

**Figure 6:**
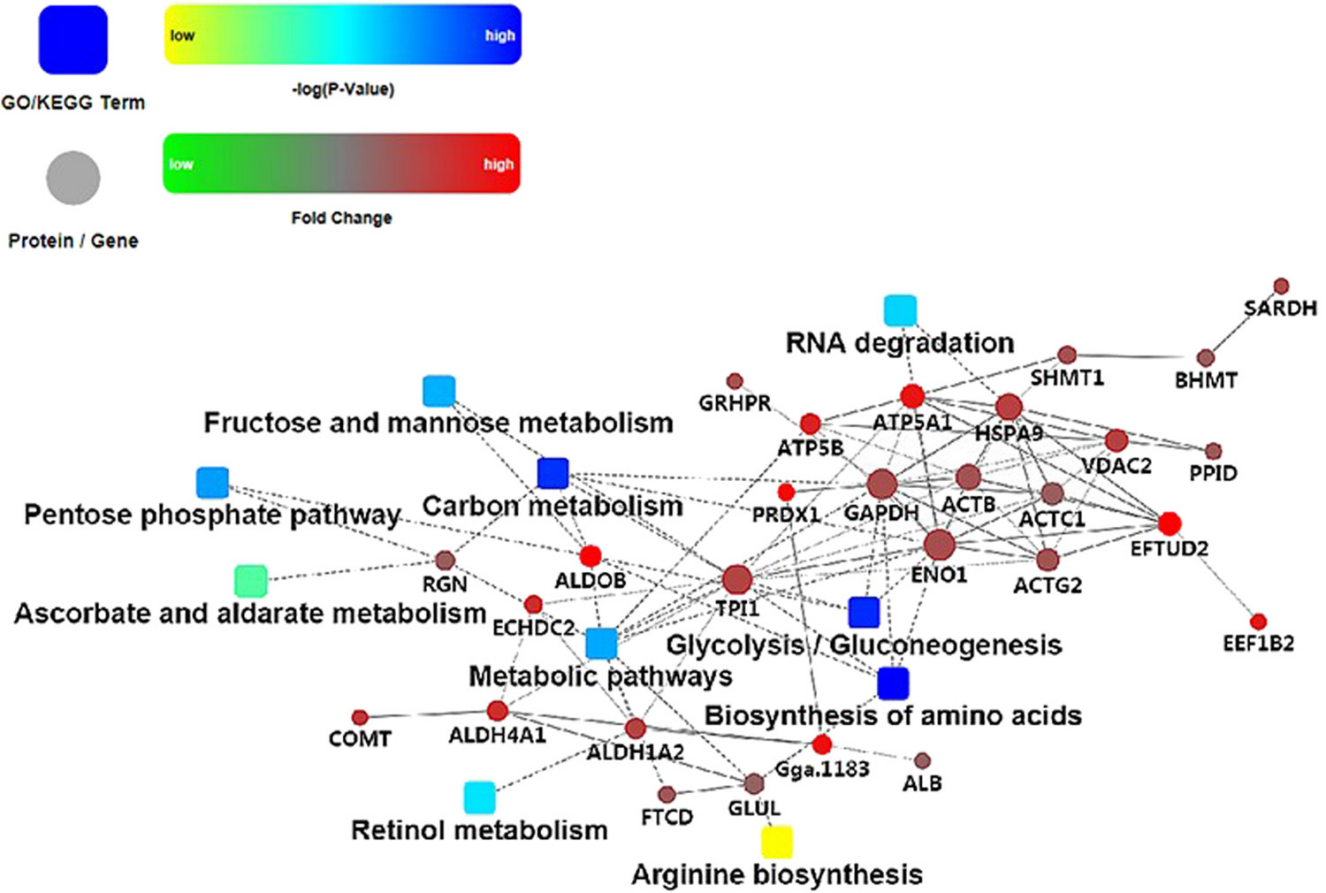
PPI from key regulatory proteins and metabolic pathways. Note: Dot represents proteins or genes, and the dot size indicates the intensity of interaction. Box represents GO or KEGG processes. Red and green are different fold changes; blue and yellow are the different log *P*-values. The solid line indicates interaction score >0.5 (dotted line indicates interaction score <0.5).

The regulatory networks of these key metabolic pathways and DEPs were analyzed. The glycolysis and gluconeogenesis pathways showed the highest degree of interaction. TPI1, ENO1, GAPDH, ALDOB, and HSPA9 proteins were of high degree. The fold changes in the expression of ALDOB and PRDX1 were the highest (Figure 6).

## 4 Discussion

Meat quality is reportedly affected by the IMF content [13]. In recent years, the meat quality of Chaohu ducks has deteriorated due to faster growth speed and shorter breeding cycles. Therefore, genetic improvements are needed to breed new varieties as per the needs of duck meat consumption. In this study, we used 2-DE combined with mass spectrometry to screen the key candidate proteins and elucidate the molecular mechanisms of lipid metabolism and fat deposition. We identified 43 DEPs between Chaohu ducks with high and low IMF, including ALDOB, GAPDH, ENO1, RGN, TPI1, HSPA9, PRDX1, and GPX1. Functional enrichment analysis revealed that DEPs were closely related to carbohydrate, lipid, and protein metabolism, including glycolysis/gluconeogenesis, pentose phosphate pathway, fructose, and mannose metabolism.

ALDOB (expressed in the liver) is one of the three isozymes of fructose-1,6-diphosphate aldolase in mammalian tissues, with the exception of ALDOA (expressed in muscle) and ALDOC (expressed in nervous tissue) [14]. Fructose-1,6-bisphosphate and aldolase mediate glucose metabolism by regulating the AMP-activated protein kinase pathway [15]. It plays a role in fructose catabolism, gluconeogenesis, and lipogenesis [16]. Enolase (*ENO1* gene code), also known as 2-phospho-d-glycerol hydrolase, is normally present in the cytoplasm and contributes to glycolysis by catalyzing the conversion of 2-phosphoglycerate to phosphoenolpyruvate in the glycolytic pathway [17]. Enolases are abundant in the cytoplasm but are also present in the cell membrane and nucleus [18]. Previous studies speculated that ENO1 promotes the accumulation of tRNA transport-binding proteins on the surface of mitochondria and is involved in the transport of aminoacyl-tRNA synthase into mitochondria, playing a regulatory role in gene transcription [19]. Enolase also participates in pyruvate synthesis from d-glyceraldehyde-3-phosphate. The expression of these two enzymes in the liver increased significantly in this study and was associated with the decomposition and transformation of carbohydrates. These enzymes also provide raw materials for fat synthesis, causing excessive fat accumulation.

Glyceraldehyde-3-phosphate dehydrogenase (encoded by *GAPDH*) plays a role in glycolysis and nuclear function. As a key enzyme in glycolysis, GAPDH catalyzes the first step of the pathway by converting d-glyceraldehyde-3-phosphate to 3-phosphate-d-glyceryl phosphate [20]. In 1957, it was demonstrated that acyl phosphatase (GAPDH) isolated from the muscle catalyzes the hydrolysis of 1,3-bisphosphoglycerate to 3-phosphoglycerate. GAPDH is also involved in cellular and cytoplasmic vesicle transport and oxidative stress [21]. Propanone phosphate isomerase (TPI1) is a highly efficient metabolic enzyme that catalyzes the conversion of dihydroxyacetone phosphate (DHAP) to phosphoglyceraldehyde during glycolysis and gluconeogenesis [22]. Studies have shown that deficiency of TPI1 is related to abnormally high levels of DHAP accumulation and oxidative stress [23]. Propanone phosphoisomerase is responsible for ATP production from glycolysis; it also produces a pyruvate molecule for conversion to glucose molecules under aerobic and anaerobic conditions, allowing rapid equilibrium of the propanone phosphoaldase produced by glycolysis. This is associated with lipid metabolism through the glycerol-3-phosphate and pentose cycles [24]. We found that these two enzymes were also significantly enriched in the glycolysis/gluconeogenesis metabolic pathway of IMF deposition, with an upstream and downstream relationship in Chaohu ducks, which confirmed the results of previous studies.

Additionally, several proteins related to lipid metabolism and oxidative stress were identified in this study. *RGN* encoding regucalcin is a calcium-binding protein that regulates intracellular Ca^2+^ homeostasis, oxidative stress, cell survival, and apoptosis [25]. Yamaguchi et al. reported that RGN is distributed in rat hepatocellular plasma and has reversible effects on the activation and inhibition of various enzymes bound to Ca^2+^ [26]. A previous study also reported that overexpression of RGN enhanced lipid accumulation in adipocyte cells, suggesting that RGN may be a novel regulator of adipocyte differentiation [27]. In this study, RGN was significantly upregulated in the CH group, which is consistent with a previous study [27].

HSPA9 is a member of the heat shock protein 70 (HSP70) family. Zhang et al. performed iTRAQ-based proteomic analysis to identify proteins in duck muscles related to lipid oxidation; they found that heat shock protein were correlated with lipid oxidation [28]. 2-DE proteomic studies showed that HSPA9 is highly expressed in the proliferation of preadipocytes of the omentum and is closely related to adipogenesis [29]. A growing number of studies have linked chaperone molecules to adipogenesis, obesity, and diabetes [30,31]. The chaperone protein HSPA9 forms a complex with the iron–sulfur cluster and uses the energy released by ATP hydrolysis to drive the conformational change and refolding of the target protein [32]. Heat shock proteins could protect lipids against reactive oxygen species (ROS)-induced lipid oxidation [33]. The overexpression of HSPA9 reduces the accumulation of ROS in glucose-deficient PC-12 cells [30]. In this study, it was found that HSPA9 was significantly upregulated in the CH group, was enriched in the process of cellular heat stress, and played an important role in the formation of mitochondrial iron–sulfur clusters.

GPX1 is a cellular and mitochondrial enzyme that catalyzes the reduction of organic hydroperoxides and hydrogen peroxide by glutathione, thereby protecting the cells against oxidative damage. Inactivation of the GPX1 gene leads to growth retardation in mice, possibly because of decreased mitochondrial energy from increased oxidative stress [34]. As a selenium-dependent enzyme that reduces the concentration of intracellular hydrogen peroxide and lipid peroxides, GPX1 plays an important role in regulating glucose homeostasis, lipogenesis, and liposis [35]. GPX1 activity is elevated in the liver and adipose tissues of pigs; it mediates lipid accumulation and fatty acid profile changes [36]. Additionally, GPX1 overexpression in mice induces elevated lipid concentrations in the plasma and tissues [37]. In this study, GPX1 was significantly enriched in several biological processes, including adipocyte differentiation and internal apoptotic signaling pathway in response to oxidative stress. Simultaneously, it was found that GPX1 was significantly upregulated, indicating the expression and activity of this enzyme in meat ducks, which is consistent with previous studies.

Peroxiredoxin 1 (encoded by *PRDX1*), a mercaptan-specific peroxidase, catalyzes the reduction of hydrogen peroxide and organic peroxides to water and alcohol, respectively. PRDX1 is a scavenger of ROS, which may be involved in signaling cascades of growth factor and tumor necrosis factor-α by regulating intracellular H_2_O_2_ concentration [38]. The expression of PRDX1 is necessary to control the response of corneal endothelial cells to agents that cause lipid peroxidation [39]. PRDX1 has a crucial role in the maintenance of lipophagic flux in macrophages [40]. Future research should investigate the implementation of protective functions, such as antioxidant stress and immunity, in the process of fat deposition and metabolism.

## 5 Conclusion

Three important pathways were identified in this study: glycolysis, gluconeogenesis, pentose phosphate pathway, and metabolism of fructose and mannose. Eight key proteins were identified, namely ALDOB, ENO1, RGN, GAPDH, TPI1, HSPA9, PRDX1, and GPX1. These proteins interact strongly with metabolic pathways and play an important role in the regulation of liver fat metabolism in Chaohu ducks. These results provide a good reference regarding the molecular mechanism of IMF deposition and fat metabolism in waterfowl for the regulation of protein expression.

## Appendix

**Table A1:** Forty-three DEPs identified by MOLDI-TOF-MS

Protein ID	Log_2_ fold change	Identity (%)	*E*-value	Uniprot-ID	Gene name
U3IHG8	3.321928095	94.78	0	P07341	ALDOB
A0A1D5PS29	3.317593505	38.95	0	Q5F3X4	EFTUD2
R0LJ39	3.063502942	98.99	5.00 × 10^−145^	P0CB50	PRDX1
U3ISH9	2.941106311	95.07	0	F1NGJ7	Gga.1183
A0A1D5PN54	2.776103988	100	0	F1NI22	ATP5A1
R0LNI3	2.627606838	98.48	1.00 × 10^−95^	F1NYA9	EEF1B2
A0A226NPQ5	2.375734539	99.19	0	Q5ZLC5	ATP5B
F1NR44	2.13093087	37	5.00 × 10^−43^	F1NSS6	ECHDC2
R0K4R7	2.084064265	92.41	0	E1C8Z8	ALDH4A1
R0KX92	1.799087306	67.79	0	O93344	ALDH1A2
R0JKM2	1.790772038	90.25	2.00 × 10^−160^	E1BTA0	COMT
R0L7N0	1.555816155	98.69	0	Q5ZM98	HSPA9
A0A1L1RLH6	1.531069493	100	0	Q9I9D1	VDAC2
A0A226MZ03	1.469885976	99.6	0	P00940	TPI1
A0A226PNR7	1.459431619	75	0	O93344	ALDH1A2
R0LLL3	1.207892852	94.22	0	E1BRE5	EEA1
F1NBJ7	1.207892852	100	0	F1NBJ7	SARDH
P19140	1.189033824	97.24	0	P51913	ENO1
A0A346M329	1.163498732	99.1	0	P00356	GAPDH
U3IR52	1.097610797	97.7	0	P51913	ENO1
G1N0B8	1.097610797	97.33	0	E1BS67	SHMT1
F1NX57	1.084064265	100	0	F1NX57	GRHPR
R0LHA7	1.042644337	100	0	P60706	ACTB
R0LBX0	1.028569152	97	0	P63270	ACTG2
U3ITD3	0.963474124	19.12	0.0009	F1NZZ2	ENSGALG00000005321
R0K8X5	0.933572638	90.24	0	F1NY09	C1H11ORF54
R0JKJ9	0.910732662	89.31	0	Q9YH58	FTCD
R0K9P5	0.910732662	80.46	0	Q8JG30	SULT1B1
R0K2N7	0.90303827	78.96	0	E1C6Z5	Gga.55096
R0LIW0	0.879705766	85.13	0	E1BV78	FGG
E1BXG9	0.879705766	100	0	E1BXG9	PPID
B4Z854	0.831877241	94.15	7.00 × 10^−117^	P07341	ALDOB
R0JHQ1	0.831877241	95.34	0	E1BZS9	APC
D2J267	0.799087306	100	4.00 × 10^−114^	R4GH86	GPX1
R0M714	0.790772038	95.29	0	E1BSH9	BHMT
R0L3A1	0.782408565	88.76	7.00 × 10^−179^	Q9I923	RGN
U3I8T6	0.722466024	99.72	0	P68034	ACTC1
R0K525	0.713695815	81.85	8.00 × 10^−151^	F1N9C1	LOC415661
T2HNS8	0.704871964	100	1.00 × 10^−112^	F1NI22	ATP5A1
R0JL78	0.678071905	92.07	0	H9KYX6	SELENBP1
R0M0W6	0.641546029	85.27	0	P19121	ALB
R0JE50	0.622930351	98.65	0	P16580	GLUL
U3J7S2	0.613531653	96.52	0	E1BYD4	NDRG1
